# A Randomized Controlled Study Comparing Patient Satisfaction and Clinical Outcomes of Different Nasal Packs in Nasal Surgery

**DOI:** 10.7759/cureus.107315

**Published:** 2026-04-18

**Authors:** Akhil P Singh, Saloni Singh, Dharmendra Kumar, Ritu Gupta

**Affiliations:** 1 Otolaryngology - Head and Neck Surgery, Sarojini Naidu Medical College, Agra, IND

**Keywords:** epistaxis management, nasal pack, nasal surgery, pain and discomfort, pain on vas

## Abstract

Introduction: Nasal obstruction is a common symptom in otorhinolaryngology, often necessitating surgical interventions that significantly impact patient quality of life. Various nasal packing materials are available, each with distinct properties influencing patient comfort and clinical outcomes. This study aimed to evaluate patient satisfaction and clinical effectiveness of three nasal packing materials, VelNez, Merocel, and Rapid Rhino nasastent, under standard surgical conditions.

Methods: A prospective, randomized, controlled, open-label, three-arm, single-center study was conducted at a tertiary medical institute after obtaining institutional ethics committee approval and registration in a national clinical trial registry. Thirty patients (18-70 years) undergoing nasal surgery were randomized into three groups: VelNez (n=10), Rapid Rhino nasastent (n=10), and Merocel (n=10). Patients were followed for 21±3 days with two follow-up visits. Outcomes assessed included hemostasis, patient comfort, and adverse events. Data were analyzed using one-way ANOVA and Student’s t-test (p ≤ 0.05).

Results: All participants completed the study, achieving effective hemostasis. Time to hemostasis was shortest with VelNez (3.9±0.92 min) compared to Rapid Rhino (9.6±0.60 min) and Merocel (9.2±0.78 min) (*p = *0.000016). VelNez had the shortest dissolution period (4.8±1.37 days) versus Rapid Rhino nasastent (12.5±1.71 days) (*p = *0.000043). Patient-reported comfort (VAS 1-10) and symptomatic relief, including pain, obstruction, and nasal discharge, were better in VelNez as compared to Merocel. No adhesions or adverse events were observed. Endoscopic Lund-Kennedy scores indicated similar healing outcomes among all three packs.

Conclusion: All three nasal packs were safe and effective. VelNez and Rapid Rhino nasastent, due to their fragmentable nature, offered improved patient comfort compared to Merocel, with VelNez showing the fastest dissolution.

## Introduction

Nasal obstruction is commonly seen in otorhinolaryngology, often causing significant distress and impacting quality of life, with an obstructed airway known to induce anxiety and even claustrophobia [1,2]. Treatment is usually surgical to improve nasal passage patency, but postoperative complications like bleeding and adhesions can occur. Various methods have been employed to manage nasal bleeds, depending on the surgeon's preference, ease of packing material placement and removal, cost-effectiveness, and patient comfort [3,4]. The postoperative period is crucial, as intranasal packing and splints help prevent synechiae and septal hematomas. Nasal packs maintain the correct positioning of the newly shaped septum and reduce the dead space between the subperichondrial layer and control bleeding [5]. In cases where intranasal packing is required, it was frequently reported by patients that the first 3-5 days following surgery are the most unpleasant [6]. Transmucosal quilting sutures have become a reliable alternative to intranasal packing after septoplasty, primarily to relieve nasal obstruction [7,8]. After surgery, patients often experienced crusting and adhesions between the middle turbinate and lateral nasal wall, leading to obstruction and symptom recurrence [9]. Nasal packs are often used to control bleeding and prevent adhesions near the middle turbinate, despite no standard treatment for these complications [10]. Nasal packing materials like VelNez, Merocel, and Rapid Rhino nasastent provide benefits, with coated polyvinyl alcohol sponges reducing mucosal trauma, preventing tissue adhesion, and lowering complications. Merocel nasal packs (Medtronic, USA) are made of compressed, dehydrated hydroxylated polyvinyl acetate sponges. These packs are highly absorbent and provide effective hemostasis by applying pressure to the nasal mucosa to control bleeding [11]. Designed to expand upon exposure to moisture, this pack exerted pressure within the nasal cavity, essential for managing bleeding. Additionally, its absorbent properties maintain a moist environment, facilitating wound healing [12]. A key advantage of Merocel over gauze is its ability to expand and absorb exudate, effectively managing postoperative nasal secretions [13-15].

The RAPID RHINO nasastent packs (Smith & Nephew, UK) are made up of carboxymethylcellulose, which promotes platelet aggregation upon contact with blood. The pack achieved hemostasis through a dual mechanism: applying pressure to control arterial bleeding while promoting clot formation to stop countless capillary and venous bleeds [16]. These packs are simple to insert and provide effective hemostasis with minimal discomfort for patients. VelNez, a biodegradable nasal pack from Datt Mediproducts Pvt. Ltd., contains gelatin, chitosan, polyvinyl alcohol, and psyllium husk [17]. The pain typically associated with traditional nasal pack removal has been completely eliminated with VelNez, as the removal is not needed due to its rapid disintegration. VelNez fragments completely and detaches itself from the compromised mucosal surfaces. Furthermore, it has been found to reduce fibrosis, promote healing, and facilitate clotting [10].

The present study was a clinical investigation conducted to compare patient satisfaction and clinical outcomes associated with three different nasal packing materials in patients undergoing nasal surgery as part of a surgeon’s routine clinical practice. Although various nasal packing materials are widely used, there are limited studies that compare clinical effectiveness and patient-centric outcomes among commonly used nasal packs. The study aimed to compare VelNez, Merocel, and Rapid Rhino nasastent nasal packs in postoperative patients for controlling bleeding, preventing synechiae, ensuring safety, and evaluating patient discomfort.

## Materials and methods

Study design

This was an open-label, interventional, comparative-controlled, three-arm, single-center study conducted at a tertiary healthcare facility. The study followed ICH-GCP and all requirements of the ICH E6(R2) Guideline for Good Clinical Practice. Participants who provided informed written consent were enrolled after approval from an institutional ethics committee (SNMC/IEC/2024/12) valid through 05/01/2024 and 04/30/2025. Informed consent was obtained in accordance with applicable regulations and ethical principles, and each participant was assigned a unique identification code to ensure confidentiality.

The study was prospectively registered in a national clinical trial registry (CTRI/2024/06/069152) and was conducted from 07/07/2024 to 13/11/2025. The trial protocol and statistical analysis plan were available under an internal protocol identification code. Participant recruitment began following regulatory and ethics approvals.

Study population and sample size

A total of 30 patients (10 per arm) were enrolled for septoplasty with or without functional endoscopic sinus surgery or turbinoplasty, with an anticipated 10% drop-out rate; study outcomes were assessed based on predefined endpoints. Patients of any gender, aged 18 to 70 years, from all racial and socio-economic backgrounds, were included if they met the study’s inclusion and exclusion criteria (Table 1). The duration of follow-up for each participant in this study was around 21±3 days.

**Table 1:** Inclusion & exclusion criteria for the study

Category	Criteria
Inclusion Criteria	1. Subject eligible for the use of a nasal pack (VELNEZ, Merocel, or Rapid Rhino nasastent) in routine clinical practice after a planned nasal surgery.
	2. Male and female subjects aged 18 to 70 years.
	3. The subject can provide written informed consent.
	4. The subject agrees to data collection at predefined follow-up periods.
Exclusion Criteria	1. Subject unable to be treated with VELNEZ, Merocel, or Rapid Rhino nasastent nasal pack in routine clinical practice after planned nasal surgery.
	2. Subject unwilling or unable to attend postoperative visits required for data collection.
	3. Subject has an active infection at the surgery site.
	4. Subject has a history of asthma.
	5. Subject is on aspirin or anti-platelet drug therapy.
	6. Subject has hypertension.
	7. Subject has a known allergy (hypersensitivity) to any of the ingredients in the nasal pack.
	8. Subject has a bleeding disorder.
	9. Any medical condition that, in the opinion of the investigator, makes the subject unsuitable (e.g., chronic, relapsing, or hereditary disease).

Randomization and study groups

**Figure 1:**
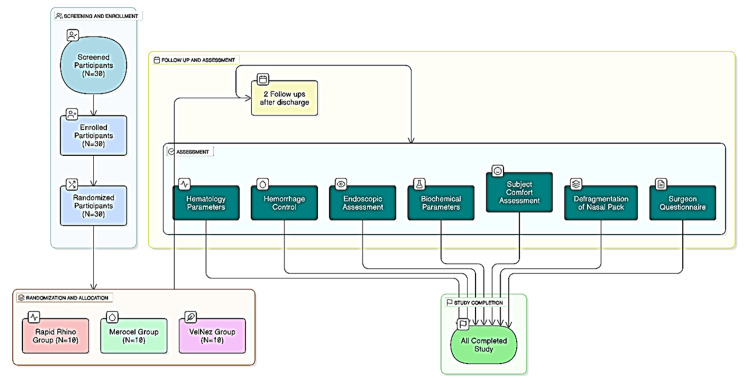
Flowchart showing design of the study

Participants were randomized into three groups by using R Software Version 4.3.0. (R Foundation for Statistical Computing, Vienna, Austria) based on simple randomization. Group A included 10 patients treated with VelNez, Group B included 10 patients treated with Rapid Rhino nasastent, and Group C included 10 patients treated with Merocel. All patients scheduled for nasal surgery requiring postoperative packing were invited to participate, providing written consent and receiving a unique ID to ensure confidentiality. The scheduling of subject visits was aligned with routine surgical practices, ensuring a minimum of three follow-up visits after discharge from the hospital, including the final visit at the end of the study (Figure 1). Each pack was sourced by the participant themselves from the local market.

Data collection and outcome measure

**Figure 2:**
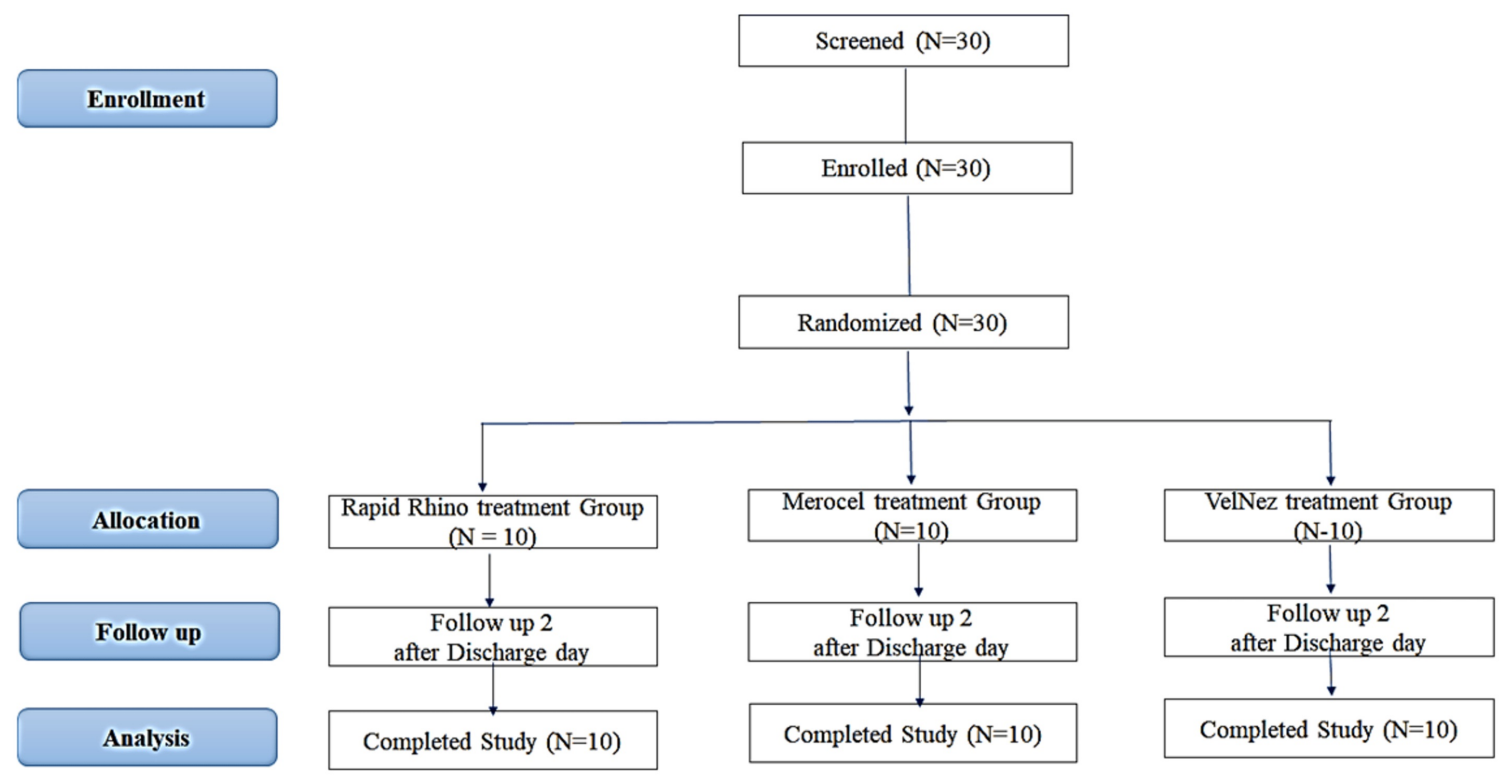
CONSORT diagram of study This flowchart shows the flow of patients during the study until the endpoint. CONSORT: Consolidated Standards of Reporting Trials

Patients enrolled in the study were evaluated on an intent-to-treat basis. A total of 30 patients scheduled for various nasal surgeries were screened and enrolled after meeting the inclusion criteria, as shown in Figure 2. The endpoint was to compare the efficacy, safety, and synechiae formation of nasal packs (VELNEZ, Merocel, and Rapid Rhino nasastent) in controlling postoperative bleeding in nasal surgery patients. All concomitant medications used during the study were recorded, including antibiotics (e.g., amoxicillin-clavulanic acid, amikacin), analgesics (paracetamol, aceclofenac), antiemetics, steroids, antihistamines, and nasal sprays. These were administered as needed for pre-existing conditions or adverse events unrelated to the study treatment. Participants were followed over a 21-day period, across five visits. Postoperative assessments were conducted at Visit 3 (Day 7 ± 3), Visit 4 (Day 14 ± 3), and Visit 5 (Day 21 ± 3), which marked the final follow-up. A comparative assessment was conducted to evaluate the efficacy of three nasal packs-VelNez, Merocel, and Rapid Rhino nasastent-in ceasing reactive bleeding and achieving secondary bleeding control in postoperative nasal surgery patients. The evaluation focused on the duration and extent of bleeding associated with each nasal pack. The assessment parameters included hemorrhage control and patient comfort, both on the day of surgery and at subsequent follow-up visits. A surgeon's questionnaire was used (1-5 scale, where 1 denoted "easy" and 5 denoted "difficult") for evaluating handling characteristics during insertion of the pack (see the Appendices). Patient-reported comfort was assessed on a 0-10 scale for symptoms such as pain, pressure, nasal discharge, dysphagia, sleep disturbance, postnasal drip, and infection. Adverse events, including infections, bleeding, and allergic reactions, were closely monitored. Synechiae were assessed by checking for adhesions between the nasal mucosa and nearby structures. A favorable endpoint was defined as the disintegration of the nasal dressing within 21 days, achievement of complete hemostasis within five minutes, reduction in pain and discomfort, and absence of infection. Endoscopic findings at each visit documented mucosal injury, middle meatal synechiae, infection, granulation, adhesion, and nasal pack migration using a grading scale (0-2). Additionally, the Lund-Kennedy scoring system was used to assess postoperative polyps, edema, and secretions and compared to baseline perioperative sinus endoscopy. Comprehensive evaluations, including physical exams, lab tests, and endoscopic assessments, were performed at baseline and final follow-up to assess safety, tolerability, and efficacy.

Statistical analysis

The results were presented as the mean ± standard deviation to ensure the accuracy of the experimental data. Statistical analysis was performed using Microsoft Excel 2010 (Microsoft Corporation, Redmond, USA). A one-way ANOVA was conducted to compare differences among the nasal packs, followed by the student’s t-test to determine statistical significance, with differences considered statistically significant at p ≤ 0.05.

## Results

The demographic parameters are summarized in Table 2.

**Table 2:** Demographic characteristics of study participants Data are presented as mean ± SD for continuous variables and N for categorical variables.

Variables	Rapid Rhino nasastent (N=10)	Merocel (N=10)	VelNez (N=10)
Age (Year)	27.10 ± 7.11	29.70 ± 8.12	29.30 ± 9.17
Male	8	5	7
Female	2	5	3
Height (cm)	166.30 ± 3.55	165.30 ± 5.24	167.20 ± 5.64
Weight (Kg)	64.18 ± 6.64	61.32 ± 7.58	66.00 ± 6.51
BMI (Kg/m²)	23.19 ± 2.08	22.43 ± 2.41	23.53 ± 1.27
Smoker	0	0	0
Non-Smoker	10	10	10

**Figure 3:**
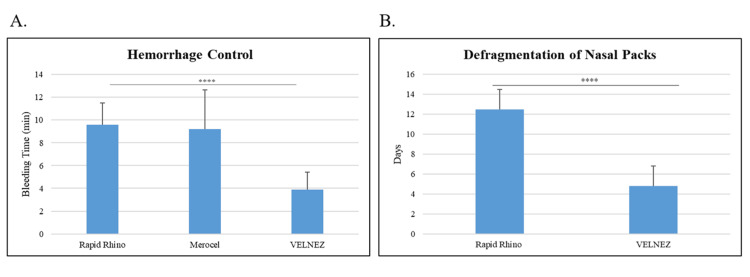
Hemorrhage control time and defragmentation of nasal pack application (A.) Hemorrhage control time and (B.) Defragmentation of Nasal pack application. ****p ≤ 0.0001. Significant p-values were obtained using Student's t-test.

Individually, all 30 patients subjected to their respective nasal applications (N=10 per group) achieved hemorrhage control. VelNez application resulted in hemostasis within 3.9 ± 0.92 minutes, whereas Rapid Rhino nasastent and Merocel achieved hemostasis in 9.6 ± 0.60 minutes and 9.2 ± 0.78 minutes (p = 0.000016), respectively (Figure 3A). The disintegration of nasal packs was also evaluated, and it was found that VelNez disintegrated within 4.8 ± 1.37 days, while Rapid Rhino nasastent took 12.5 ± 1.71 days (p = 0.000043), as depicted in Figure 3B.

**Figure 4:**
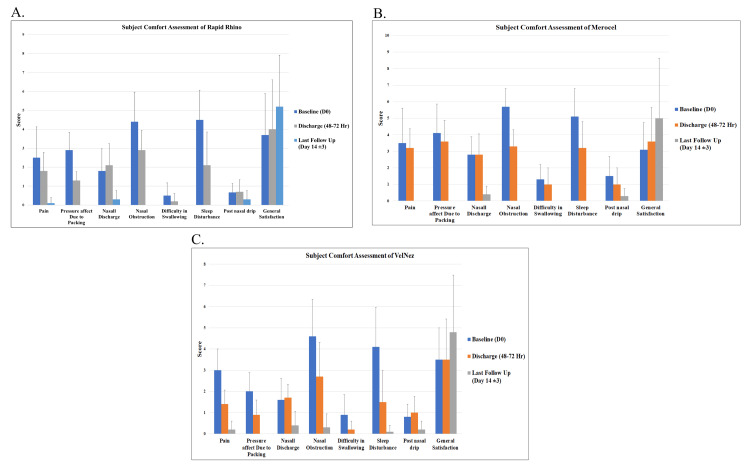
Graphical representation of patient comfort with each nasal pack Graphical representation of subject comfort assessment in patients treated with nasal packing materials. (A) Rapid Rhino, (B) Merocel, and (C) VelNez groups. Subject comfort was evaluated at baseline (Day 0), at discharge (48–72 hours), and during follow-up visits until the end of the study. The study endpoint for each patient was determined at the clinician’s discretion based on recovery status, treatment response, and overall health condition.

In contrast, Merocel required manual removal, and it was removed at 48 to 72 hours post-surgery. Secondary bleeding was observed only in the Merocel group, while no cases were observed in the Rapid Rhino nasastent and Velnez groups. Rapid Rhino and Velnez were associated with lower pain scores and reduced bleeding compared to Merocel. As Merocel is a highly absorbent material, it absorbs fluid within approximately 30 seconds. In comparison, Rapid Rhino nasastent takes around 15 minutes for fluid absorption, while Velnez absorbs fluids in a maximum of 30 seconds, similar to Merocel. Subject comfort assessment showed that from discharge to the last follow-up, VelNez provided symptom relief comparable to Rapid Rhino nasastent and Merocel. All assessments were evaluated on the scale (VAS) of 1-10. In the case of the VelNez application, pain reduced from 1.40±0.66 to 0.20±0.40, nasal obstruction from 2.70±1.62 to 0.30±0.64, and nasal discharge from 1.70±0.64 to 0.40±0.49. As far as sleep disturbance was concerned, it dropped from 1.50±1.53 to 0.10±0.30, aligning with Rapid Rhino nasastent and Merocel. Overall, all three packs demonstrated effective comfort improvement, matching the performance of other nasal packs (Figures 4A-4C).

**Figure 5:**
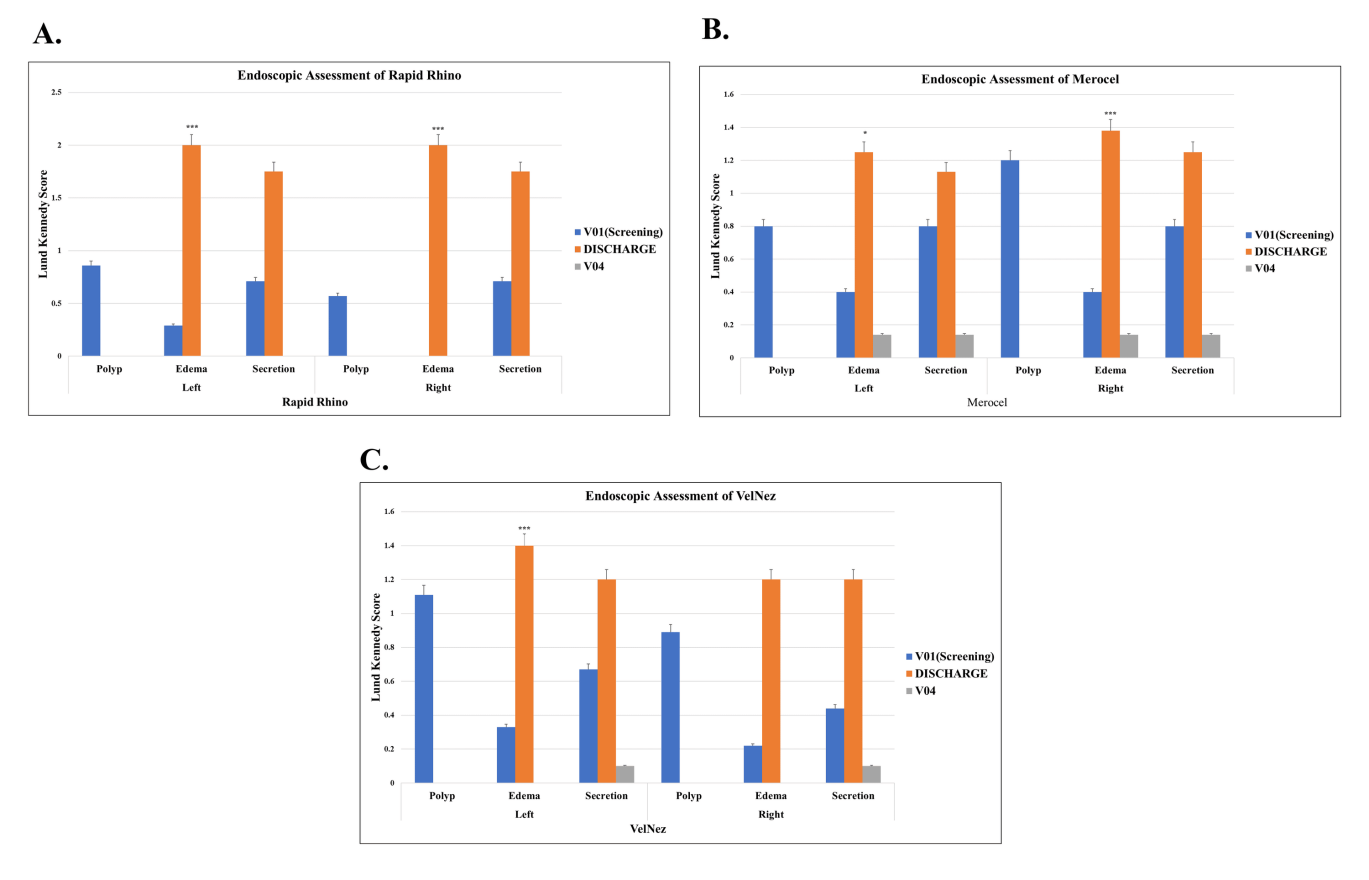
Graphs showing endoscopy assessment of nasal packs Graphs showing Endoscopy assessment of nasal packs (A) Rapid Rhino, (B) Merocel, and (C) VelNez using Lund-Kennedy and POSE scores. *p ≤ 0.05, ****p ≤ 0.0001. Significant p-values were obtained using one-way ANOVA. POSE: Perioperative sinus endoscopy

None of the patients reported any problems related to these parameters till the end of the study. No adhesions were observed in any subject after surgery with nasal pack application, and no adverse events were reported during the study. The endoscopy assessment using Lund-Kennedy scores showed that all three nasal packs demonstrated similar healing outcomes by the final follow-up. Edema scores at discharge were highest in Rapid Rhino nasastent (2.00±0.00), followed by VelNez (1.40±0.80 left, 1.20±0.75 right) and Merocel (1.25±0.66 left, 1.38±0.70 right), but all groups showed complete resolution by the final follow-up. By the last follow-up, secretion levels had almost completely resolved in all groups, with VelNez showing minimal residual secretion (0.10±0.30), comparable to the others (Figure 5).

Statistically, all groups showed significant improvements (p < 0.05) at discharge, and by the final visit, the differences were non-significant, indicating similar recovery patterns. During the study, blood samples were collected for hematological analysis, including RBC count, WBC count, platelet count, and the measurement of cholesterol, glucose, and liver enzymes (Table 3).

**Table 3:** Hematological parameters of patients at screening and the last follow-up visit on Day 4 Data are presented as mean ± SD. Continuous variables were compared between groups using one-way ANOVA, with corresponding test statistics and p-values reported where applicable. The end of the study for each patient was determined at the clinician’s discretion based on recovery status, treatment response, and overall health condition.

	Screening Day				Visit 4				
	Merocel	Rapid Rhino nasastent	VelNez	p-value	Merocel	Rapid Rhino nasastent	VelNez	p-value	Biological reference range (unit)
Hemoglobin (gm/dl)	12.47±2.21	13.92±2.00	12.44±2.13	0.22	13.1±1.52	14.08±1.61	12.61±2.10	0.88	11.5-15 (gm/dl) (F), 13-17 (gm/dl) (M)
Total RBC count (million cells/mm^3^)	4.02±0.64	4.643±0.73	4.108±0.61	0.09	4.33±0.51	4.15±0.52	4.004±0.91	0.30	3.8-4.8 (million cells/mm³)
Total WBC count (cells/mm^3^)	10330 ± 4119.61	8630±2372.08	6630±1140.22	0.02	8080±1815.86	8920±1934.37	8320±1852.81	0.78	4000-10000 (cell/mm³)
Platelet count (lakh cells/mm^3^)	2.14±0.72	1.881±0.58	2.266±2.14	0.80	2.40±0.87	2.13±0.88	2.66±1.84	0.77	1.50-4.50 (lakh cells/mm³)
Neutrophil (%)	67.3±10.36	66.8±7.83	66±10.75	0.95	69±9.66	67.3±8.60	69.18±8.02	0.66	40-80 (%)
Eosinophil (%)	3.5±1.96	4±3.16	3±0	0.58	3±0.47	3.1±0.32	3.56±2.15	0.49	1-6 (%)
Lymphocytes (%)	30.2±15.07	27.3±8.47	29±10.75	0.85	26±9.66	27.5±8.57	46.5±67.04	0.51	20-40 (%)
Monocytes (%)	2±0.47	1.8±0.67	2±0	0.49	2±0.47	2.1±0.32	2.2±0.42	0.64	2-10 (%)
Hematocrit (packed cell volume)	37.08±6.27	41.45±6.75	36.71±7.21	0.20	36.41±9.00	36.04±9.43	34.06±8.36	0.27	36-46%
Mean corpuscular volume (MCV) (fl)	91.1±11.61	90.12±9.10	89.51±6.77	0.03	88.5±5.87	87.71±5.22	83.51±4.59	0.49	80-100 (fl)
Mean corpuscular hemoglobin (MCH) (pg)	30.46±3.01	29.9±3.18	30.26±2.07	0.35	30.78±2.71	29.81±1.72	28.06±1.75	0.54	27-32 (picogram)
Mean corpuscular hemoglobin concentration (MCHC) (g/dl)	33.55±2.44	33.24±0.91	33.449±1.68	0.63	34.55±2.07	33.73±0.93	33.78±1.76	0.25	32-35 (gm/dl)
RBC dispersion width (%)	15.77±1.41	14.73±1.45	15.35±2.36	0.12	15.3±1.95	15.03±1.54	15.19±1.63	0.48	11.5-14.5%

The results showed no significant changes in most parameters, which remained within the normal range, except for SGOT levels in patients treated with Merocel (p = 0.39), which were above normal limits. Additionally, in some cases, LDL/HDL levels were found to be elevated in patients treated with Rapid Rhino nasastent (p = 0.22) (Table 4).

**Table 4:** Biochemical parameters monitored on screening day and last follow-up visit Data are presented as mean ± SD. Continuous variables were compared between groups using one-way ANOVA, with corresponding test statistics and p-values reported where applicable.

Dressing Applied	Screening Day				End of the study				
	Merocel	Rapid Rhino	VelNez	p-value	Merocel	Rapid Rhino	VelNez	p-value	Biological reference range (unit)
Total bilirubin (mg/dl)	0.55±0.33	0.552±0.20	0.503±0.16	0.87	0.67±0.49	0.65±0.25	0.53±0.96	0.65	0.3-1.2 (mg/dl)
SGOT (IU/L)	50.562±27.30	63.357±72.55	42.04±23.62	0.59	51.47±43.16	32.31±15.91	43.47±28.13	0.39	5-40 (IU/L)
SGPT (IU/L)	61.911±51.50	65.878±79.57	39.178±21.77	0.52	51.42±32.56	30.13±20.71	54.14±42.51	0.22	5-42 (IU/L)
ALKP (IU/L)	226.69±59.33	224.83±52.85	244.56±106.05	0.82	212.75±37.86	198.85±56.37	217.98±62.64	0.96	60-290 (IU/L)
Creatinine (mg/dl)	1.144±0.64	1.225±0.44	1.166±0.20	0.92	1.043±0.38	0.99±0.29	1.103±0.61	0.87	0.4-1.4 mg/dl
BUN	NA	NA	NA		NA	NA	NA	-	7-20 (mg/dl)
Random blood sugar (mg/dl)	97.89±25.68	100.167±21.09	105.19±18.15	0.91	104.96±26.41	105.27±25.60	104.51±19.85	0.99	70-140 (mg/dl)
Total cholesterol (mg/dl)	150.9±30.17	171.286±26.64	156.71±43.13	0.39	154.40±41.30	168.26±19.37	144.84±32.20	0.28	130-200 (mg/dl)
HDL cholesterol-direct (mg/dl)	50.06±22.23	57.661±22.46	47.51±8.72	0.46	47.04±9.06	48.09±5.60	43.58±8.33	0.41	40–60 mg/dL
LDL cholesterol-direct (mg/dl)	61.1±25.20	68.92±20.71	82.04±34.37	0.24	76.6±27.02	84.66±18.82	71.8±22.13	0.45	100–129 mg/dL (near optimal)
Triglycerides (mg/dl)	184.00±96.26	163.63±79.13	170.19±115.5	0.90	146.65±72.18	134.01±61.43	140.34±65.25	0.91	150–199 mg/dL
TC/HDL cholesterol ratio	3.20±0.81	3.76±2.99	3.22±0.59	0.74	3.27±0.46	3.4675±0.55	3.29±0.34	0.59	3.5–5.0 (moderate risk)
LDL/HDL ratio	1.61±0.89	1.917±2.61	1.97±0.71	0.90	9.70±25.41	1.78±0.46	1.65±0.39	0.38	2.0–4.0 (moderate risk
VLDL cholesterol (mg/dl)	36.467±18.84	34.62±17.11	24.49±14.29	0.25	31.5±12.83	47.54±24.77	35±22.18	0.20	5–30 mg/dL
NON-HDL cholesterol	NA	NA	NA	NA	NA	NA	NA	NA	30–159 mg/dL

**Figure 6:**
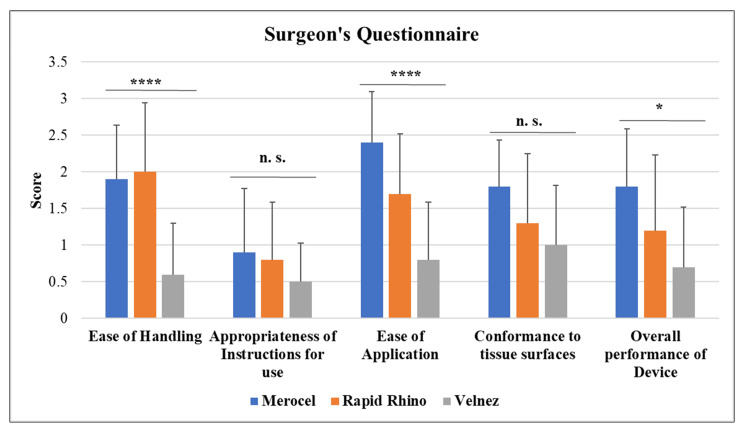
Surgeon's questionnaire with responses captured on a 5-point Likert-type scale evaluating surgeons' experiences with the usage of nasal pack Surgeons rated the devices on a 1–5 Likert scale, where 1 denotes “easy” and 5 denotes “difficult.” The presented data were obtained from three independently conducted experiments. n. s. Non-significant, *p ≤ 0.05, ****p ≤ 0.0001. Significant p-values were obtained using one-way ANOVA.

Surgeons’ experiences with nasal packs were evaluated using a Likert-scale questionnaire to assess overall effectiveness. Statistical analysis confirmed the significance of the data, indicating reliable differences in performance among the nasal packs. VelNez demonstrated significantly higher overall performance in ease of handling, ease of application, and conformance to tissue surfaces. This was likely due to its pliability. Rapid Rhino nasastent showed moderate performance, while Merocel ranked the lowest, being the hardest to apply due to its unyielding shape. VelNez is the most acceptable choice, followed by Rapid Rhino nasastent, with Merocel being the least favorable, as reported by the surgeon (Figure 6). From patients’ perspective also, VelNez and rapid Rhino nasastent were the most comfortable packing material when compared to Merocel.

## Discussion

A perfect nasal pack should effortlessly adapt to the shape of the nasal passages and reliably promote blood clotting. It should be easy to insert and remove, comfortable, and stay securely in place without slipping. Furthermore, it should successfully stop bleeding without harming the nasal lining and result in minimal tissue irritation. Several techniques have been employed to reduce the pain associated with nasal pack removal, such as intramuscular papaveretum injection and other methods [18]. The packing material should create a healing-friendly environment, minimize tissue damage, and lower infection risk. Non-absorbable (NAS) packs like gauze, Merocel, Vaseline, and balloon tamponades control bleeding by pressure but may eventually damage the mucosa. To minimize compression-induced mucosal trauma, surgeons may cover the nasal pack with a latex finger from surgical gloves, use a special coating to prevent adhesions, or employ a saline-inflated balloon to control bleeding while minimizing damage. Resorbable nasal packs achieve hemostasis by utilizing either coagulating factors or stimulators. Although several studies have compared various types of absorbable (AS) nasal packs, both among themselves and with the commonly used removable packs, no clear consensus has been established regarding the superiority of any specific packing material. These concerns can be addressed through the use of biodegradable and fragmentable nasal packs. Our study found that VelNez achieved controlled hemostasis, i.e., 3.9±0.92 minutes, better than Rapid Rhino nasastent and Merocel. In contrast, previous studies reported that Surgiflo, a sterile AS porcine gelatin, achieved intraoperative hemostasis within 10 minutes of product application [19]. Recent studies revealed that the hemorrhage control time for VelNez was 7.49 ± 3.90 minutes in the study population in post-nasal surgery (Bellad et al., 2024) [10]. As far as the Rapid Rhino nasastent was concerned, it has shown significantly less bleeding with respect to the Merocel (p < 0.05) [20]. Merocel is a widely used NAS nasal packing material made of compressed, dehydrated hydroxylated polyvinyl acetate. However, as a NAS solid, it may cause pain and bleeding during removal, nasal obstruction, and mucosal edema [21]. In contrast, VelNez degrades naturally, eliminating painful removal while ensuring effective hemostasis. Its self-fragmenting property enhances patient comfort and improves postoperative outcomes in nasal surgery. As far as postoperative discomfort is concerned, previous studies reported that Merocel and gauze packs had the highest pain ratings during removal [22]. However, in the current study, both VelNez and Rapid Rhino nasastent applications reduced pain, nasal obstruction, and nasal discharge. A potential limitation of the study is its open-label design, which may have influenced subjective outcomes such as patient-reported comfort and symptom relief. While objective measures such as hemostasis and endoscopic healing were unaffected, patient expectations may have introduced bias in the assessment of comfort and satisfaction. Our study shows that VelNez, Merocel, and Rapid Rhino nasastent nasal packs are equally effective for postoperative healing, with comparable outcomes in polyp resolution, edema reduction, and secretion control. Our findings are consistent with prior research on nasal packing materials, where it was previously reported that silastic splints were effective in minimizing synechiae formation [23]. Several studies have compared AS and NAS spacers, such as Merocel, Nasopore, and MeroGel, in terms of postoperative outcomes. Other studies report lower synechiae rates with NAS Merocel packing than with saline irrigation alone (11.3% vs. 53.7%, p < 0.05) [24,25]. Earlier studies found that AS MeroGel and NAS Merocel had comparable synechiae rates, with a tendency for the MeroGel group to get more interventions. On the other hand, none of the individuals in our study had intranasal adhesions.

Limitations of the study

This study has several limitations that should be considered while interpreting the results. First, the sample size was relatively small, with only 30 participants included in total. Such a limited sample reduces the statistical power of the study and may affect the generalizability of the findings to a broader patient population. Second, the study compared three different nasal packing materials, of which two were biodegradable and one was non-biodegradable. The unequal distribution between biodegradable and non-biodegradable categories may limit the ability to draw strong comparative conclusions, specifically between these two groups of materials. Third, the short duration of follow-up may not fully capture long-term outcomes, delayed complications, or differences in mucosal healing associated with the different nasal packing materials. Additionally, the study may be subject to potential observer and patient-reported bias due to its open-label design, particularly in subjective outcomes such as pain, discomfort, or nasal obstruction. Finally, as the study was conducted in a single clinical setting with a limited number of participants, the results may not be fully representative of outcomes in different institutions, surgical techniques, or patient demographics. The study was conducted at a single center, which further limits external validity and generalizability, and multicenter validation is warranted. Larger, multicentric studies with longer follow-up are required to validate and strengthen the findings.

## Conclusions

Hemostasis occurred fastest with VelNez, while Rapid Rhino nasastent and Merocel required substantially longer times to achieve bleeding control. VelNez and Rapid Rhino, being biodegradable, reduced discomfort from pack removal and improved patient satisfaction. In summary, this study suggests that VelNez, Merocel, and Rapid Rhino nasastent are all effective and well-tolerated choices for nasal packing.

## Appendices

**Table 5:** Surgeon's questionnaire

Surgeon's Questionnaire		
S. No.	Question	Response
1.	Ease of handling	☐ 0 (Easy) ☐ 1 ☐ 2 ☐ 3 ☐ 4 ☐ 5 (Difficult)
2.	Appropriateness of instruction for use	☐ 0 (Easy) ☐ 1 ☐ 2 ☐ 3 ☐ 4 ☐ 5 (Difficult)
3.	Ease of application	☐ 0 (Easy) ☐ 1 ☐ 2 ☐ 3 ☐ 4 ☐ 5 (Difficult)
4.	Conformance to tissue surfaces	☐ 0 (Easy) ☐ 1 ☐ 2 ☐ 3 ☐ 4 ☐ 5 (Difficult)
5.	Overall performance of the device	☐ 0 (Easy) ☐ 1 ☐ 2 ☐ 3 ☐ 4 ☐ 5 (Difficult)
